# Preclinical Model of Stereotactic Ablative Lung Irradiation Using Arc Delivery in the Mouse: Is Fractionation Worthwhile?

**DOI:** 10.3389/fmed.2021.794324

**Published:** 2021-12-24

**Authors:** Annaïg Bertho, Morgane Dos Santos, Sarah Braga-Cohen, Valérie Buard, Vincent Paget, Olivier Guipaud, Georges Tarlet, Fabien Milliat, Agnès François

**Affiliations:** ^1^Laboratory of Radiobiology of Medical Exposures, Institute for Radioprotection and Nuclear Safety (IRSN), Research Department in Radiobiology and Regenerative Medicine, Fontenay-aux-Roses, France; ^2^Laboratory of Radiobiology of Accidental Exposures, Institute for Radioprotection and Nuclear Safety (IRSN), Research Department in Radiobiology and Regenerative Medicine, Fontenay-aux-Roses, France

**Keywords:** radiation, lung, stereotactic body radiation therapy, fibrosis, mouse, fractionation

## Abstract

Lung stereotactic body radiation therapy is characterized by a reduction in target volumes and the use of severely hypofractionated schedules. Preclinical modeling became possible thanks to rodent-dedicated irradiation devices allowing accurate beam collimation and focal lung exposure. Given that a great majority of publications use single dose exposures, the question we asked in this study was as follows: in incremented preclinical models, is it worth using fractionated protocols or should we continue focusing solely on volume limitation? The left lungs of C57BL/6JRj mice were exposed to ionizing radiation using arc therapy and 3 × 3 mm beam collimation. Three-fraction schedules delivered over a period of 1 week were used with 20, 28, 40, and 50 Gy doses per fraction. Lung tissue opacification, global histological damage and the numbers of type II pneumocytes and club cells were assessed 6 months post-exposure, together with the gene expression of several lung cells and inflammation markers. Only the administration of 3 × 40 Gy or 3 × 50 Gy generated focal lung fibrosis after 6 months, with tissue opacification visible by cone beam computed tomography, tissue scarring and consolidation, decreased club cell numbers and a reactive increase in the number of type II pneumocytes. A fractionation schedule using an arc-therapy-delivered three fractions/1 week regimen with 3 × 3 mm beam requires 40 Gy per fraction for lung fibrosis to develop within 6 months, a reasonable time lapse given the mouse lifespan. A comparison with previously published laboratory data suggests that, in this focal lung irradiation configuration, administering a Biological Effective Dose ≥ 1000 Gy should be recommended to obtain lung fibrosis within 6 months. The need for such a high dose per fraction challenges the appropriateness of using preclinical highly focused fractionation schedules in mice.

## Introduction

Thoracic radiation therapy exposes healthy lung tissue to ionizing radiation, responsible for the development of radiotherapy-associated side effects such as radiation-induced pneumonitis and fibrosis (1–3). Historical preclinical models of conventional radiotherapy-associated toxicity include whole thorax and hemi-thoracic irradiation in mice and rats. Since the pioneering studies by Travis et al. (4, 5), these models have paved the way for significant advances in the understanding of tissular, cellular and molecular mechanisms of radiation pneumonitis and fibrosis (6).

Therapeutic management of early stage lung tumors has radically changed in recent years, with the use of stereotactic body radiation therapy (SBRT) for the treatment of early stage non-small cell lung cancer [NSCLC (7)]. SBRT includes changes in two main parameters in radiation therapy schedules compared to conventional radiotherapy: significantly reduced irradiated target volume and severe hypofractionation. Thanks to adapted or dedicated small animal irradiation devices generating image-guided highly focused beams with submillimetric precision (8), several studies have been published describing the consequences of radiation exposure of limited lung volumes in mice. Previously reported data used different collimated beams, ranging from 1 mm diameter to 10 × 10 mm and a broad range of single doses from 10 to 120 Gy. The lung has a large reserve capacity and the volume exposed to ionizing radiation is a strong determinant of its functional tolerance (9). Unsurprisingly, the larger the exposed volume is, the lower the possible dose that can be delivered without generating high animal mortality (10–12). Using 3 × 3 mm beam collimation, which seems to be the most suitable for modeling SBRT in mice (11, 13–16), the dose delivered in one single fraction can be as high as 120 Gy (and possibly higher) without associated animal mortality (12), providing that the delivery methods include multiple beam entries to avoid life-threatening thoracic skin/muscle lesions. This highlights the strong volume impact when considering lung response to radiation exposure. Moreover, several authors argue in favor of considering that specificities exist in the response to limited lung volume exposure, such as different patterns of serum cytokine changes (16) or specific gene and protein expression following small field exposure compared to large field (17).

In addition to changes in lung target volume, SBRT is characterized by the use of severe hypofractionation. With its parallel organization, the lung is known to be a good candidate for such changes in fractionation schedules (18). However, the impact of such severe hypofractionation associated with reduced exposed volume on the normal lung merits further investigation. All previously published data on limited lung volume exposure has used single doses, except for three studies. The work of Du et al. (19) demonstrated the effective targeting of spontaneous lung tumors in a genetically-engineered mouse model using 3 weekly fractions of 20 Gy with 5 × 5 mm beam collimation. The authors did not report any normal tissue damage in this configuration, while pointing out that experiments stopped 8 weeks post-exposure, probably too short a time for fibrosis to develop. Delivering 3 × 6.67 Gy to the mouse lung using 5 × 5 mm beam collimation induced changes in lung tissue density but no change in the Ashcroft score within 26 weeks post-exposure (20). Finally, the study by Soysouvanh et al. showed that no lung fibrosis occurred following 5 fractions of 20 Gy using 3 × 3 mm collimated beam even 15 months post-exposure (21).

With a view to opening up the possibilities for future research, the question we addressed in this study work was as follows: in incremented models of preclinical SBRT exposure with reduced target volumes, is it worth using fractionated protocols or should we continue focusing solely on volume limitation? In this study, we show that using a 3 fractions/1 week regimen, a dose per fraction of at least 40 Gy is necessary for the development of lung fibrosis within 6 months, a reasonable time lapse given the mouse lifespan. No dose-effect was seen for the parameters measured, suggesting a threshold effect for lung fibrosis development. The need for such a high dose per fraction challenges the appropriateness of using preclinical highly focused fractionation schedules in mice.

## Materials and Methods

### Animals and Irradiation Procedure

Male C57BL/6JRj mice from Janvier Labs (France) were used for all experiments. Animals were housed in the IRSN animal facilities accredited by the French Ministry of Agriculture for performing experiments on rodents. Animal experiments were performed in compliance with French and European regulations on the protection of animals used for scientific purposes (EC Directive 2010/63/EU and French Decree 2013–118). All experiments were approved by Ethics Committee #81 and authorized by the French Ministry of Research under the reference APAFIS#13021-2018011217442982 v1 (internal project number P17-13).

Irradiation was performed on the SARRP (Small Animal Radiation Research Platform, XSTRAHL Ltd., UK) using arc therapy. The muriplan treatment planning system was calibrated by Xstrahl during the commission of the SARRP platform. For that, the reference dose rate was measured in dose to water with a cylindrical ionization chamber and depth doses using EBT3 radiochromic films for each collimator were performed. After this calibration carried out by the company, we have completed the characterization of the SARRP by making Half Value Layer measurements (HVL) and reference dosimetry measurements with ionization chamber following the AAPM TG61 protocol (22). Moreover, dose profiles using EBT3 films were also performed at different depths for each collimator. For the 3 × 3 mm irradiation field at 1 cm depth, the dose rate is about 2.3 Gy/min in dose to water. Daily, to ensure the proper functioning of the platform and the proper delivery of the beam, reference dose rate measurements (AAPM TG61protocol) are performed with an ionization chamber. A maximal deviation of 1% from the reference value is accepted. Each month, quality controls concerning positioning and targeting are also carried out.

For irradiation, mice were anesthetized using 100 mg/kg ketamine (Imalgene 1000) and 10 mg/kg xylazine (Rompun^®^ 2%, Bayer Healthcare, France) to create profound anesthesia and limit respiratory motion as far as possible. Anesthetized mice were immobilized on a restraint treatment bed and the isocenter was placed on the left lung on the CBCT image using the treatment planning system Muriplan^®^, by taking anatomical landmarks for reference. CBCT images were obtained using an uncollimated beam (20 × 20 cm), a high voltage of 60 kV, an intensity of 0.8 mA with an inherent and additional filtration of 0.8 and 1 mm of beryllium and aluminum respectively, with continuous beam on and 360° (horizontal) stage rotation between the x-ray source and the digital flat panel detector. A total of 236 projections were obtained and a 3D reconstruction image of the mouse was transferred to the Muriplan^®^ dose planning, verification and delivery system. After image segmentation into air, lung, fat, tissue and bone, we then placed the isocenter within the left lung. Irradiations were performed at 220 kV and 13 mA with inherent and additional filtrations of about 0.8 and 0.15 mm of beryllium and copper respectively.

The left lung was exposed to 3-fraction irradiation schedules (Monday, Wednesday and Friday), with different doses per fraction, i.e., 20, 28, 40 or 50 Gy, delivering total doses of 60, 84, 120 or 150 Gy respectively. The fractionation schedules were chosen based on the results obtained by Lavigne et al. (23). Indeed, following arc-delivered 3 × 3 mm collimated beam of 90 Gy to the mouse lung, Lavigne et al. observed that decreased lung capacity was significantly impacted by lung volumes receiving 30 Gy or more, i.e., 30% of the prescribed dose. Regarding the strong volume impact on lung damage, we decided, for the purposes of our fractionation strategy, to track the Biological Effective Dose (BED) received at the 30% isodose. BED was calculated as follows: D[1+(d/α/β)]; with D as total dose, d as dose per fraction and α/β as alpha/beta ratio. We thus applied an α/β ratio of 3 Gy for normal lung and constructed four fractionation schedules focused on a BED at the 30% isodose close to 100 Gy (108 Gy, 3 × 28 Gy), corresponding to the limit generally accepted for normal tissue in clinical practice, a BED <100 Gy (61 Gy, 3 × 20 Gy) and two BED values higher than 100 Gy, i.e., 208 and 304 Gy for 3 × 40 and 3 × 50 Gy, respectively. These fractionation protocols gave BED values to the isocenter of 460, 868, 1720, and 2650 Gy for 20, 28, 40, and 50 Gy per fraction, respectively.

An arc therapy treatment using a 3 × 3 mm collimator with immobile stage and 220° gantry rotation from −110 to 110° was planned for each fraction. To be able to irradiate the same region on the left lung, CBCT images of the first fraction were co-recorded and manually superimposed over the day's CBCT images.

### MicroCT Imaging

Mice were anesthetized by inhalation of 1.5% isoflurane. Mouse lung imaging was performed using two micro-CT scanners (Quantum FX and Quantum GX2, PerkinElmer) with respiratory gating. On the Quantum FX, images (performed at the *Plateforme d'imagerie du vivant, Université Paris Descartes, Faculté de Chirurgie Dentaire*) were acquired at 90 kV, 160 μA, no additional filtration and with a field of view of about 20 × 20 × 20 mm for a resolution of 40 μm. The estimated shooting time was about 4 min and 30 s and 512 slices were acquired. The mean dose absorbed by the mice was about 1653 mGy. On the Quantum GX2 (IRSN), images were acquired at 90 kV, 88 μA, with an additional filtration of 0.06 mm of copper + 0.5 mm of aluminum and with a field of view of about 36 mm with a reconstruction of 25 mm allowing for a resolution of 50 μm. The estimated shooting time was about 4 min and 512 slices were acquired. The mean dose absorbed by the mice was about 900 mGy of dose to water.

The images were analyzed using AnalyzePro Software, which allows for semi-automatic and manual segmentation. The main bronchi and right and left lungs were segmented using the semi-automatic option with the same threshold range for each mouse in order to be able to compare them. Lesions induced by irradiation–the injury patch–were manually contoured by the same operator. From these segmentations, volumes were computed and, for each volume, Hounsfield Unit (HU) histograms were extracted using a bin width of 1-HU. For density distribution representation, smoothing curves were constructed using PRISM software applying second-order smoothing with 60 neighbors.

### Lung Tissue Treatment

Studies were performed 6 months post-exposure. Given the small size of the irradiated area, different animals were dedicated to histological analyses and mRNA preparations. For histology, the right and left lungs were fixed in 4% paraformaldehyde and embedded in paraffin. Five-micrometer paraffin tissue sections were used for HES and Masson's trichrome staining and for immunohistological studies. For the purposes of mRNA preparation, the irradiated area, ipsilateral and contralateral lung tissues were frozen in an RNAlater^TM^ RNA Stabilization Reagent (Qiagen, CA) pending analysis. To avoid variations in the structural/cellular constitution of different areas of the lung, age-matched control/unirradiated mice were included for lung imaging and histological analyses and measurements were performed in matched areas between irradiated and non-irradiated mice.

### Histopathology and Immunohistochemistry

HES and Masson's trichrome-stained tissue sections were used to assess overall lesion severity. The thickness of the alveolar septa was measured using the Visiol@b^TM^2000 image analysis software (Biocom SA, France). Immunostaining of club cells (uteroglobin, UGB) and type II pneumocytes (prosurfactant protein C, SFTPC) was used to monitor parenchymal and epithelial lung damage.

UGB-positive cells were counted on the bronchiolar epithelium both inside and far from the injured area, taking into consideration at least three different bronchioles. UGB-positive cells were expressed as a percentage of total cells. The total number of cell nuclei per 100 μm was also tracked in order to monitor radiation-induced variations in total epithelial cell density. SFTPC-positive cells were counted both inside and far from the injured area with between 3 and 5 different fields (X 400 magnification).

### Tissue RNA Extraction and Quantitative Real-Time PCR

Total RNA was prepared using the mirVana isolation kit (Thermo Fisher Scientific, France). After quantification on a NanoDrop ND-1000 apparatus (NanoDrop Technologies, DE), reverse transcription was performed with 1 μg of RNA using a reverse transcription kit from Applied Biosystems (France). Relative mRNA was quantified using the ΔΔCT method with 18S as the housekeeping gene. Genes were chosen as lung cell markers or according to their proven role in lung tissue inflammatory response to radiation exposure.

### Statistics

Data are expressed as means ± SEM. Statistical analyses were performed by ANOVA (Tukey multiple comparison *post-hoc* test) or Student's *t*-test, with a level of significance of *p* < 0.05.

## Results

### Lung Fibrosis at 6 Months Is Obtained for 3 × 40 Gy or More

**Figure 1:**
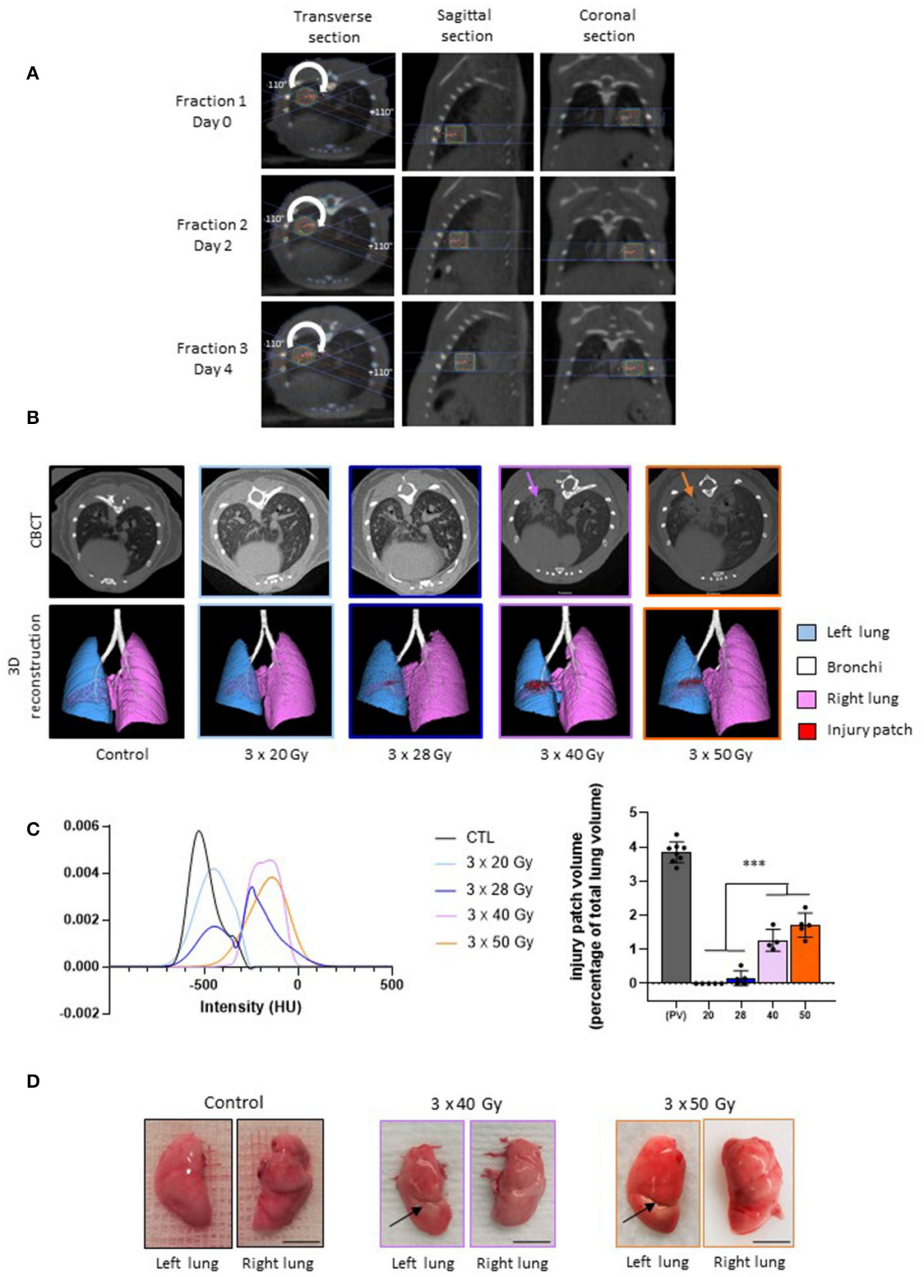
Lung fractionated stereotactic arc therapy generates long-term lung fibrosis. **(A)** Treatment planning and repositioning for fractionated dose to the left lung in mice. Representative dose distribution of each fraction performed at Days 0, 2, and 4, with Day 0 as the day of the first fraction. Arc-therapy was applied from −110° to +110° (arrow). Images are given for the 3 × 40 Gy schedule. **(B)** Micro-computed tomography and 3-dimensional reconstructions performed in control mice and 6 months post-exposure to three fractions of 20, 28, 40, and 50 Gy irradiation. Arrows indicate focal radiation-induced lung opacification. **(C)** Left panel: smoothed mean intensity representation, in Hounsfield Units (HUs), in the patch (or in the corresponding area) from groups of mice exposed to 3 × 20, 28, 40 or 50 Gy compared to control values; right panel: volume of the injury patch and expressed as a percentage of the total lung volume compared to the calculated planned volume (PV) exposed to ionizing radiation. For irradiated tissues, colors are the same as in the left panel. ***p < 0.001 compared to the target volume (TV) group. **(D)** Example of macroscopic observation of left and right lungs in control mice and 6 months following 3 × 40 and 3 × 50 Gy. Arrows indicate radiation-induced stricture. *n* = 4–8 per group. Scale bar represents 5 mm.

Focal fractionated irradiation can be achieved successfully using the scanner image performed for the first fraction as a repositioning standard before each next fraction (Figure 1A). At 6 months post-irradiation, a well-defined injury patch was detectable on the CBCT imaging using 40 and 50 Gy per fraction only (Figure 1B, upper line). Three-dimensional reconstruction confirmed well-defined severe tissue damage at these doses per fraction (Figure 1B, lower line, in red on the image). Lung tissue opacification was confirmed by mean smoothed HU curves showing an increased intensity of the patch (Figure 1C, left panel, mauve and orange curves). After 28 Gy per fraction, three mice showed slight opacification and two mice showed no injury patch when observing the CBCT images. Three-dimensional reconstruction gives an example of slight tissue damage when observable (Figure 1B, lower line). The HU curve for this schedule (dark blue) showed two peaks: the left peak corresponding to mice with no detectable opacification and the right peak to those developing detectable opacification. No sign of tissue damage was ever observable using CBCT or 3D reconstruction following 20 Gy per fraction. The patch volume calculation confirmed significant patch development after 40 and 50 Gy per fraction (Figure 1C, right panel). Conversely, no significant difference was observed between 20 and 28 Gy per fraction, confirming the small size of patches developed in the three mice concerned after 28 Gy per fraction. Note that the patches are smaller than the theoretical exposed volume due to tissue stricture and consolidation. Macroscopic observation showed white stricture across the left lung indicative of severe radiation-induced tissue scarring for 40 and 50 Gy per fraction (Figure 1D, arrows). No such tissue consolidation was observed following 20 and 28 Gy per fraction schedules.

**Figure 2:**
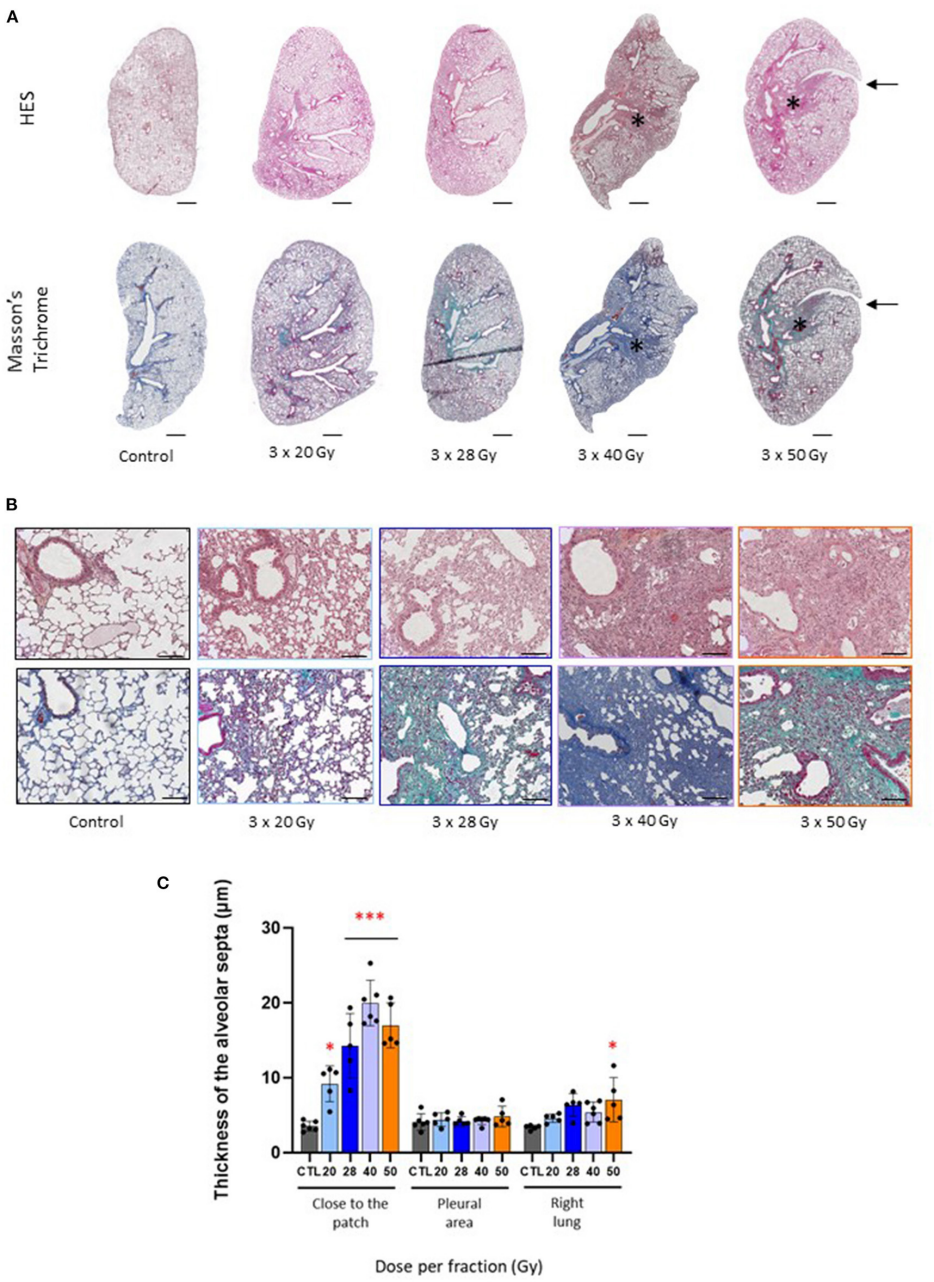
Fraction size of 40 Gy or higher generates lung tissue fibrosis after 6 months. **(A)** Images of HES and Masson's trichrome stained control and focally irradiated left lungs 6 months following 3 × 20, 28, 40 or 50 Gy. Asterix and arrows indicate radiation-induced fibrosis and stricture, respectively. Scale bar represents 1 mm. **(B)** Details of lung parenchymal damage induced by 3 × 20, 28, 40, and 50 Gy either following HES (upper line) or Masson's Trichrome staining (lower line). Scale bar represents 200 μm. **(C)** Thickness of the alveolar septa was measured close to the injury patch, far from the patch (near the pleura) and in the right lung 6 months following focal exposure to 3 × 20, 28, 40 or 50 Gy. Measurements were performed in corresponding areas for control tissues. *p < 0.05; ***p < 0.001 compared to control group. *n* = 5 or 6 per group.

Both 40 and 50 Gy per fraction schedules generated tissue fibrosis, as observed on left lung histological sections (Figure 2A, asterisk), showing dense radiation-induced tissue damage. For 3 × 28 Gy, a cartography of a mouse displaying slight opacification on CBCT is given as an example, but there is no well-defined injury patch compared to 40 and 50 Gy per fraction. Note the stricture visible on the top right of the 3 × 50 Gy cartography (arrows). Focal fibrosis was obtained only following the 40 and 50 Gy per fraction schedules, but all protocols generated significant parenchymal injury (Figure 2B) with a reactive increase in the thickness of the alveolar septa (Figure 2C).

### Lung Fibrosis Is Associated With Loss of Club Cells and Increased Type II Pneumocyte Numbers

**Figure 3:**
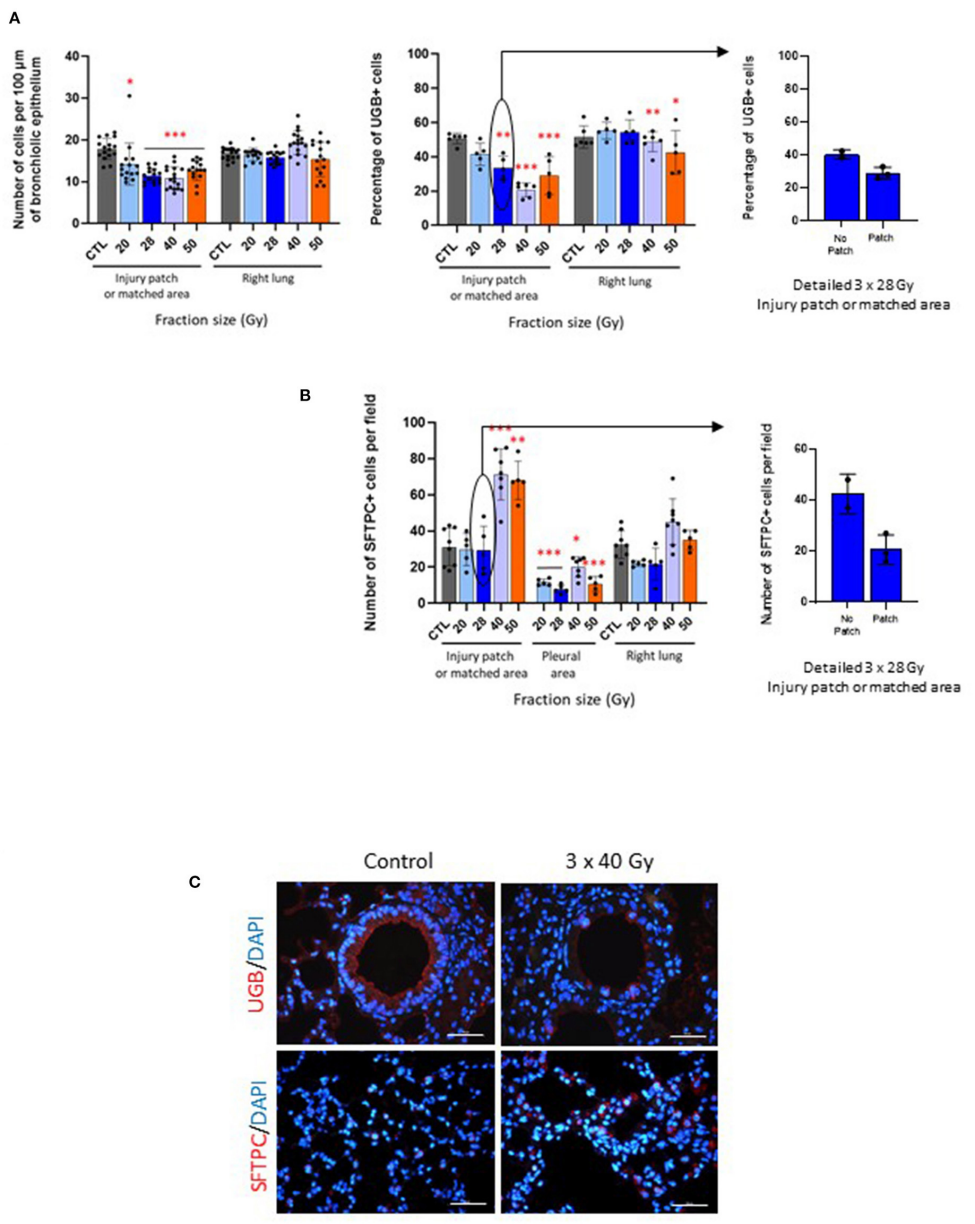
Fibrosing schedules induce club cell loss and increased numbers of type II pneumocytes. **(A)** Number of cells per 100 μm of bronchiole epithelium (left-hand graph) and percentage of UGB-positive cells (middle graph) measured in the injury patch or matched area and right lungs in control and focally irradiated tissues 6 months after 3 × 20, 28, 40 or 50 Gy. Right-hand graph shows detailed data for 3 × 28 Gy-exposed mice with or without visible patch. *p < 0.05; **p < 0.01; ***p < 0.001 compared to respective control groups. *n* = 5 or 6 per group, except for number of cells: 1 point = 1 bronchiole. **(B)** Number of SFTPC-positive cells per field counted in the injury patch or matched area, far from the patch near the pleural area and in the right lung 6 months after 3 × 20, 28, 40 or 50 Gy and in control tissue (left-hand graph). Right-hand graph shows detailed data for 3 × 28 Gy-exposed mice with or without visible patch. *p < 0.05; **p < 0.01; ***p < 0.001 compared to respective control groups. *n* = 5–8 per group. **(C)** Images of uteroglobin and pro-surfactant protein C immunostainings (UGB, marker of club cells and SFTPC, marker of type II pneumocytes, respectively) in control tissues and after 3 × 40 Gy schedule as an example. Scale bar represents 100 μm.

All fractionation schedules were associated with a significantly decreased number of cells per 100 μm of bronchiolar epithelium measured in the injury patch or matched area, with a lesser effect following 20 Gy per fraction (Figure 3A, left-hand graph). No effect was seen in the right lung. Schedules with doses per fraction of 28, 40, and 50 Gy induced a loss of club cells, with repercussions in the right lung for 40 and 50 Gy per fraction (Figure 3A, middle graph). Interestingly, the 3 mice exposed to 3 × 28 Gy displaying a slight injury patch showed a tendency to a lower number of club cells than those having no patch at all, bearing in mind that, with only two or three animals, this cannot be statistically tested (Figure 3A, right-hand graph).

Both the 40 and 50 Gy per fraction schedules induced increased type II pneumocyte numbers, with decreased numbers in the pleural area for all schedules (Figure 3B, left-hand graph). No significant change was observed in the right lung. The three mice displaying an injury patch after 3 × 28 Gy exposure showed a tendency to have fewer SFTPC-positive cells than those displaying no patch (with reservations given that statistical analyses are not possible for club cell numbers), but all mice remained similar to the control mice, as indicated in the previous graph (Figure 3B, right-hand graph).

Figure 3C shows images as examples of club cells and type II pneumocytes immunostainings in control and 3 × 40 Gy exposed lungs.

### Gene Expression Levels Are Not Influenced by Fraction Size

**Figure 4:**
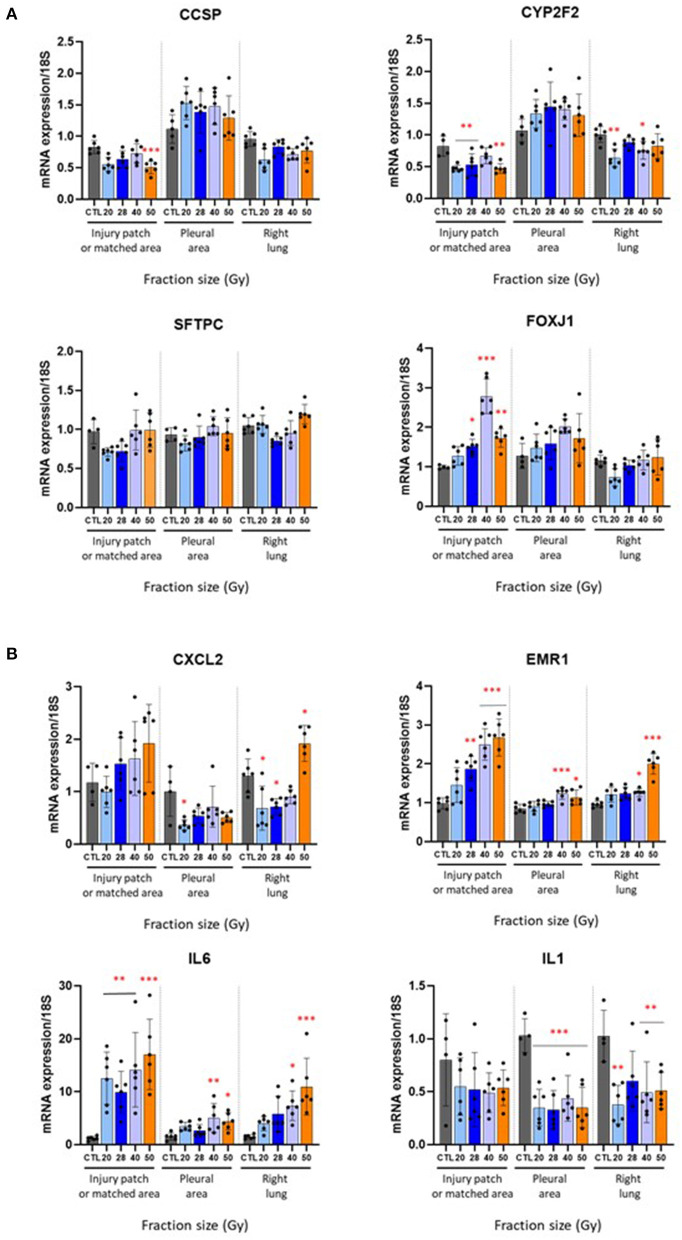
Effect of different fractionation schedules on mRNA expression. Changes in gene expression levels in the injury patch or matched area, pleural area, and right lung 6 months after radiation exposure to 3 × 20, 28, 40 or 50 Gy. **(A)** Club cell (CCSP, CYP2F2), type II pneumocytes (SFTPC), and ciliated cell (FOXJ1) markers. **(B)** Inflammatory mediators CXCL2, EMR1, IL6, and IL1. *p < 0.05; **p < 0.01; ***p < 0.001 compared to respective control groups. *n* = 4–6 per group.

Overall, lung cell markers were not strongly affected by irradiation at this time point and followed the club cell and type II pneumocyte numbers shown in Figure 3. Ccsp and Cyp2f2 (club cell markers) were reduced after fractions of 50 Gy only, and 20, 28, and 50 Gy respectively (Figure 4A). SFTPC remained at control levels in the patch, ipsilateral and contralateral lungs. Foxj1 (ciliated cell marker) increased in the patch following radiation exposure to 3 × 28, 40, and 50 Gy. Gene expressions of classic inflammatory markers (Cxcl2, Emr1, Il6, and Il1) were very slightly influenced by fraction size (except Emr1). Note several repercussions on the ipsilateral and contralateral lungs. The profiles are shown in Figure 4B.

### A Biological Effective Dose (BED_3Gy_) of 1000 Gy May Be Considered as a Threshold for Focal Lung Fibrosis Development Within 6 Months in Our Irradiation Configuration

**Figure 5:**
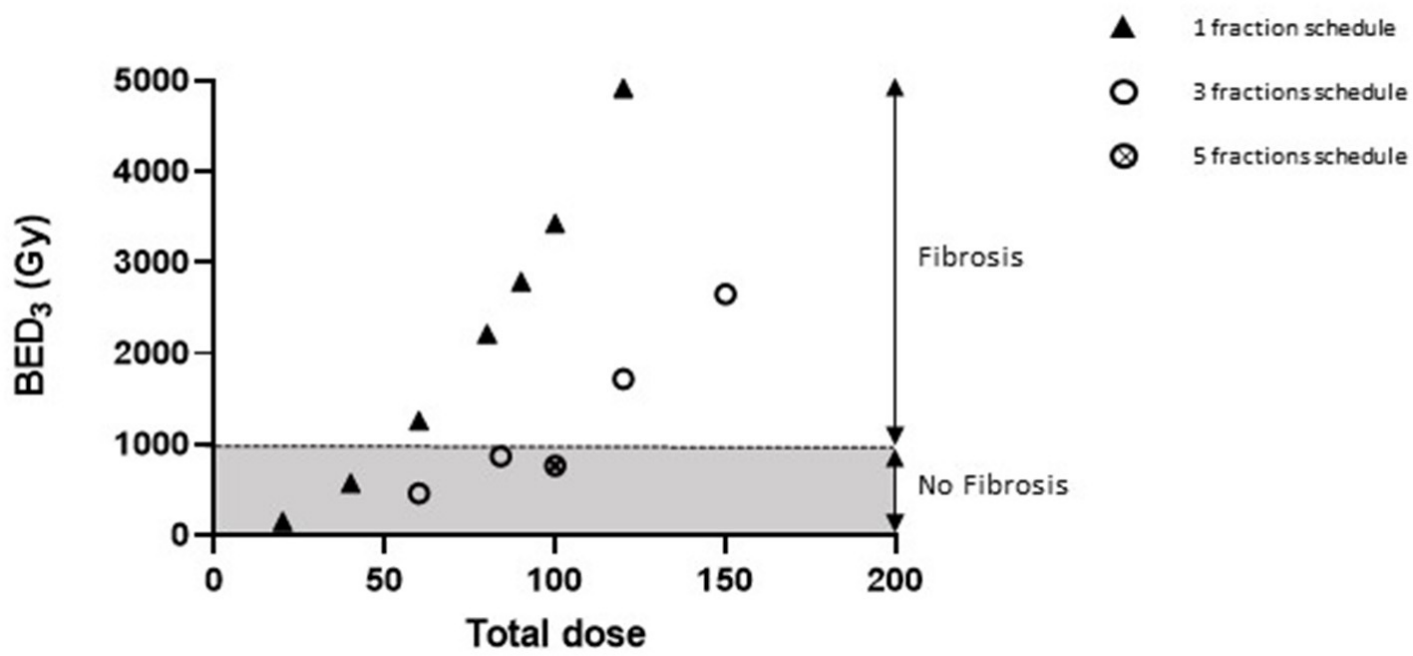
A BED of 1000 Gy would be necessary for focal lung fibrosis development. Graphic representing the association between BED values (considering α/β ratio = 3 Gy for healthy lung tissue) and lung fibrosis development or otherwise within 6 months of irradiation. Data were obtained after arc therapy using 3 × 3 mm beam collimation from this study (three fractions schedules), from Lavigne et al. (23) (90 Gy single dose), Soysouvanh et al. (21) (90 Gy single dose and 5 × 20 Gy schedule), and Bertho et al. (12) (20–120 Gy single doses).

The association of the BED value with different total doses delivered following various schedules used in this study, as well as for previous publications from our laboratory, shows that a BED_3Gy_ of 1000 Gy (considering α/β ratio = 3 Gy for a healthy lung) seems to be a threshold for focal lung fibrosis development within 6 months (Figure 5).

## Discussion

The aim of this study was to determine whether or not it is worth using fractionated schedules in the context of SBRT preclinical modeling, compared, where possible, to what has previously been published in the literature relative to single doses.

The first challenge involved the choice of fractionation schedules. We based our choice on previous observations. To begin with, schedules with three fractions were chosen to limit uncertainties of animal repositioning during multiple fractions. Delivering five fractions generates diffuse injured areas and difficulties in damage contouring on CBCT images (21). A single study time-point at 6 months post-exposure was kept as a representative and relatively long period for lung fibrosis to develop, while remaining reasonable given mouse lifespan, and thus avoiding mouse aging and spontaneous lung tumor development. Before this time-point of 6 months and during the inflammatory period, injury is difficult to evaluate and contour using CBCT, resulting in imaging which is not particularly informative given the objective of this study. It should be borne in mind that the results of our study are valuable if we consider this time period of 6 months, but that it cannot be excluded that waiting longer may allow fibrosis to be observed. Finally, following arc-delivered/3 × 3 mm beam collimation of 90 Gy to the mouse lung, Lavigne et al. observed that decreased lung capacity was significantly impacted by lung volumes receiving 30 Gy or more, i.e., 30% of the prescribed dose (23). We thus decided to construct fractionation schedules giving BEDs to the 30% isodose close to 100 Gy (108 Gy for 3 × 28 Gy), corresponding to the limit generally admitted for normal tissue in the clinic, a BED <100 Gy (61 Gy for 3 × 20 Gy) and two BED values higher than 100 Gy, i.e., 208 and 304 Gy for 3 × 40 and 3 × 50 Gy, respectively. We are aware that the choice of α/β ratio and BED calculation using the linear quadratic model (LQM) is controversial (24–26). It has been suggested that LQM is not appropriate for small dose (<1 Gy) and high dose (>10 Gy) exposures (25). Although some publications do show that LQM may be used in the range of 15–20 Gy (26), studies are still needed to confirm the relevance of using LQM in SBRT or to construct another model that can be used in the clinic, taking into account not only radiation-induced cell killing but also damage to the vascular and stromal systems (27). Last, it should be noted that Klement et al. recently concluded that it might be possible for clinical practitioners to use LQM to compare different fractionation schedules in SBRT for NSCLC, even if the α/β ratio for tumors may be greater than is generally accepted (28, 29). Nevertheless, and insofar as regards our study, conscious of the limitations of the model, we decided to use LQM anyway, knowing that our objective was to compare high doses per fraction and single dose schedules only, rather than make a comparison with conventional fractionation schedules. Hopefully, the future will tell us if such decisions were appropriate or not.

In this study, we show that administering 3 × 20 Gy does not lead to lung fibrosis when considering a period of 6 months. This schedule may be representative of some fractionation strategies used in clinical practice (30). This may be surprising considering that historical and ongoing publications on radiation-induced lung fibrosis demonstrate that doses of 15/20 Gy on the whole thorax are sufficient to induce lung fibrosis (6, 31–33). This illustrates the strong volume impact encountered in parallel-organized organs such as the lung. Several studies used a single dose of 20 Gy, leading to fibrosis or not, depending on the volume exposed: using 7-mm-diameter field induced collagen deposition within 6 months (13), 5-mm circular parallel-opposed fields showed radiation-induced lung fibrosis at 39 weeks (34) and parallel opposed/5 × 5 mm-collimated beams generated lung fibrosis within 6 months (35). Interestingly, the Ashcroft score (26 weeks) following 20 Gy/5 × 5 mm-beam irradiation (20) was lower than following 15 Gy whole thorax exposure [20 weeks (36)]. Using the same 5 × 5 mm-beam collimation and 7 to 10 non-overlapping and non-opposing beams to target spontaneous lung tumors with 3 weekly fractions of 20 Gy, Du et al. did not observe any effect on normal tissue (19). In addition to the probably too early time point (8 weeks) mentioned by the authors, a smaller irradiated volume (beams target the tumor) may explain the lack of effects on normal tissue. We previously observed that using smaller beams (3 × 3 mm-collimated) did not induce lung fibrosis following a single dose of 20 Gy (12). In this study, and even delivering 3 × 20 Gy, a reduced irradiated volume and a probably lower mean lung dose (35) are responsible for the lack of lung fibrosis development, as observed by Soysouvanh et al., using the same beam arrangements and delivering 5 × 20 Gy (21).

A clinically relevant fractionation schedule of 3 × 20 Gy, exposing around 4% of the total mouse lung volume, is not sufficient to generate lung fibrosis in 6 months. When dose per fraction was increased, we obtained what we may call “not convincing” results regarding 3 × 28 Gy, which could appear as a threshold fractionation schedule, leading to lung fibrosis in some mice only. This needs to be confirmed, probably using a greater number of animals, for any definite conclusions to be drawn. Schedules using 40 and 50 Gy per fraction induced lung fibrosis in 6 months. The absence of dose-response effect relative to the parameters measured in this study (morphometrical measures, cell counting and gene expression levels) may suggest the notion of threshold. Only fibrosing schemes were associated with significant club cell depletion, as previously observed following single dose focal lung exposure (12). Club cells play an important role in lung epithelial homeostasis and repair (37). The absence of club cells compromises epithelial repair and favors peribronchiolar fibrosis (38), and chronic club cell depletion can be used as a model of lung fibrosis (39). Club cell depletion is also associated with 15 or 17 Gy radiation exposure to the whole thorax (32, 40) and plasma CCSP (club cell secretory protein) can be used as a marker for adverse radiation outcome and, especially, for fibrosis development in fibrosis-prone C57 mice [5 Gy total body + 10 Gy to the whole lung (41)], suggesting in this and in previously published work that loss of club cells may signal fibrosis development (12). Type II pneumocytes also show stem cell properties and participate in lung alveolar space in the case of injury (42, 43) and targeted injury of type II pneumocytes is sufficient to induce pulmonary fibrosis (44). Senescence, apoptosis and epithelial to mesenchymal transition (EMT) of type II pneumocytes may play a role in fibrosis development (45, 46). However, the data remains controversial, with both decreased (45, 47) or increased numbers following both single or fractionated doses (32, 48, 49). In general, less is known about club cells and type II pneumocyte numbers following focal radiation exposure. Does the need for much higher doses to obtain significant club cell depletion and fibrosis reflect the possibility that surviving club cells will colonize damaged tissue from the margins? Is the increased number of type II pneumocytes beneficial to the lung or does this favor EMT? This merits further investigation.

As mentioned above, we are aware that BED calculation remains controversial for very high doses per fraction or single doses (25, 50). However, it may be observed that, in this study, protocols giving rise to tissue fibrosis within 6 months present similar BEDs to previously published studies using 3 × 3 mm collimation and arc therapy, i.e., probably above 1000 Gy (taking an α/β ratio of 3 Gy for a healthy lung) (12, 21, 23). In this case, however, we had to use very high doses per fraction to induce tissue fibrosis. The range of doses seems to be as dissimilar to clinical practice as the single doses used in previous publications mentioned above, raising questions as to the translational relevance of such models. Moreover, we observed some repercussions outside the irradiation field, as reported by Ghita et al. (35) and Lavigne et al. (23), describing acute ipsilateral red hepatization following 90 Gy focal exposure. This was particularly illustrated in the present study by changes in gene expression levels in the pleural area and ipsilateral lung, mainly occurring at higher doses per fraction. This suggests that there may be non-targeted effects but also an unintended dose received by neighboring tissue. This implies that the ipsilateral lung may not be considered as unirradiated, and thus justifies the use of independent control mice.

## Conclusion

If we consider a reasonable number of fractions and overall treatment time, the fractionation schedules that can be used in preclinical models of SBRT with the objective of inducing tissue fibrosis within a reasonable time lapse cannot be more representative of clinical practice than previously published single doses. Considering that whole and hemi-thorax exposures already used doses in the ablative range, these new models of focal radiation exposure will influence tissue response more by volume reduction than by changes in the doses delivered and/or the schedule applied. We will probably have to accept being far from the schedules/doses used in a clinical setting and will probably have to consider volume-related clinical relevance rather than dose/schedule-related relevance. The remaining questions tend toward tumor-bearing models. Does a schedule exist that could be used to target a lung tumor while irradiating a sufficient volume of normal tissue, allowing enough time for side effects to develop? This warrants further exciting study.

## Data Availability Statement

The original contributions presented in the study are included in the article/supplementary materials, further inquiries can be directed to the corresponding author.

## Ethics Statement

The animal study was reviewed and approved by Ethics Committee #81 and French Ministry of Research under the reference APAFIS#13021-2018011217442982 v1.

## Author Contributions

AF and FM contributed to the conception and design of the study. MD constructed the model. AB, MD, SB-C, VB, GT, and AF collected and interpreted the data. AF and AB wrote the first manuscript draft. FM, MD, VP, and OG realized critical review of the manuscript. All authors contributed to manuscript revision, read, and approved the submitted version.

## Funding

This work was supported by the ROSIRIS program, with funding from IRSN, Cancéropôle d'Ile de France and INCA, Institut National du Cancer (INCa 2018-1-PL BIO-06-).

## Conflict of Interest

The authors declare that the research was conducted in the absence of any commercial or financial relationships that could be construed as a potential conflict of interest.

## Publisher's Note

All claims expressed in this article are solely those of the authors and do not necessarily represent those of their affiliated organizations, or those of the publisher, the editors and the reviewers. Any product that may be evaluated in this article, or claim that may be made by its manufacturer, is not guaranteed or endorsed by the publisher.
